# Depression and social support among breast cancer patients in Addis Ababa, Ethiopia

**DOI:** 10.1186/s12885-019-6007-4

**Published:** 2019-08-27

**Authors:** Abigiya Wondimagegnehu, Workeabeba Abebe, Aynalem Abraha, Solomon Teferra

**Affiliations:** 1 Department of Preventive Medicine, School of Public Health, College of Health Sciences, Addis Ababa University, Addis Ababa, Ethiopia; 2 Institute of Medical Epidemiology, Biostatistics and Informatics, Martin-Luther-University, Halle, Germany; 3 Department of Pediatrics and Child Health, School of Medicine, College of Health Sciences, Addis Ababa University, Addis Ababa, Ethiopia; 4 Department of Oncology, School of Medicine, College of Health Sciences, Addis Ababa University, Addis Ababa, Ethiopia; 5 Department of Psychiatry, School of Medicine, College of Health Sciences, Addis Ababa University, Addis Ababa, Ethiopia; 6 Harvard T.H. Chan School of Public Health, Harvard University, Boston, USA

**Keywords:** Breast cancer, Depression, Social support

## Abstract

**Background:**

Depression is a common co-morbid, disabling disorder that affects 10–25% of cancer patients. It causes substantial functional impairment and lowers survival rate of breast cancer patients. Therefore, the aim of this study is to determine the magnitude of depression and its association with social support among breast cancer patients in Addis Ababa, Ethiopia.

**Methods:**

A cross-sectional study which included 428 breast cancer patients was conducted in seven health facilities in Addis Ababa, Ethiopia. Depression and Social Support were assessed using standard tools Patient Health Questionnaire 9 (PHQ 9) and Multidimensional Scale of Perceived Social Support (MSPSS) respectively. Descriptive statistics were done based on the standard PHQ9 cut off points (0–4, 5–9, 10–14, 15–19 and ≥ 20). Mann-Whitney and Kruskal Wallis tests were employed to compare MSPSS score among depressed and non-depressed patients and across the different levels of depression. Bivariate and multivariate logistic regression was done to identify factors associated with depression.

**Result:**

The prevalence of depression among breast cancer patients was 25% (107/428), andaccording to the PHQ9 score categorization, 70/428 (16.4%), 30/428 (7.01%) and 7/428 (1.64%) of these patients were having moderate, moderately severe and severe depression respectively. Age, occupation, type of health facility treated, severity of pain, hormonal therapy and having problem with employer/ family were significantly associated with depression. The participants’ MSPSS total score was overall found to be high (70.35 ± 16.81). Those women who had moderate and severe depression had lower mean MSPSS scores compared to women with none/ minimal depression (*P* = 0.002).

**Conclusion:**

This study found that one in four breast cancer patients had depression. Depression is associated with poor social support given by family, friends and significant others. Therefore, screening for depression and psychosocial service should be integrated in the routine breast cancer care in Ethiopia.

**Electronic supplementary material:**

The online version of this article (10.1186/s12885-019-6007-4) contains supplementary material, which is available to authorized users.

## Background

Breast cancer is one of the leading cancers in developing countries with an estimated 882,900 new cases and more than 324,300 deaths each year. An estimated 63,100 breast cancer deaths occur in Africa annually [1, 2]. Based on 2013 data from the Addis Ababa Cancer Registry, breast cancer accounted for 31.4% of all cancer cases [3], and several other studies done in different parts of Ethiopia also revealed breast cancer as the most prevalent cancer in Ethiopia [4–7]. According to the World Health Organization (WHO) country profile report in 2018, breast cancer is the leading cancer in Ethiopia with an estimated 15, 244 (22.6%) new cases and 5 years prevalence of 46.7%. Despite the fact that breast cancer is mostly curable disease in developed countries, Ethiopia is one of the countries with highest age standardized mortality rate which is reported to be 22.9 per 100,000 population [8].

Depression is a comorbid disabling disease that affects approximately 15 to 25% of cancer patients [9]. Several epidemiological studies revealed that depression is very common among breast cancer patients which is associated with substantial functional impairment and diminished survival [10]. According to reports from various studies, the prevalence of depression among breast cancer patients ranged from 9.3 to 56% [10–14]. A recently conducted systematic review which included several studies all over the world reported that the prevalence of depression among breast cancer patients is 32.2% [15].

Patients with breast cancer and comorbid depression have worse pain, fatigue, seriously impaired quality of life and even diminished overall survival [13, 14, 16, 17]. A recent meta-analysis found a 25% higher mortality for cancer patients with depressive symptoms and a 39% higher mortality for those with major depression after adjusting for other prognostic factors [18]. Some studies reported that psychotherapeutic and social support for patients with breast cancer was found to be associated with improved survival [18, 19]. In addition to survival benefits of psychosocial support, other studies also recently revealed that it had a great role in improving quality of life [20, 21] and significantly reducing level of depression and other common mental disorders [21–24].

Evidence suggests that social support has a great impact in reducing depression score among breast cancer patients. However, depression is not properly screened and appropriate social support is not being given for breast cancer patients in developing countries. Although there are few studies done on breast cancer in Ethiopia, most of them deal with other aspects of the disease like overall magnitude, knowledge, attitude and practice, quality of life and survival [4, 7, 25, 26]. However, there is no data on the overall burden of depression among breast cancer survivors in Ethiopia and the association between social support and depression in this population has not been adequately explored. Therefore, this study was conducted to determine the magnitude of depression among patients with breast cancer and it might contribute evidence to support screening for depression and integration of psychosocial service in the routine cancer care in Ethiopia.

## Methods

### Study design and place

The study was conducted between December 2017 – May 2018. A cross sectional study design was used to assess the magnitude of depression among breast cancer patients in seven health facilities in Addis Ababa, Ethiopia. Ethical clearance was obtained from the Research Ethics Committee (REC) of School of Public Health and Institutional Review Board (IRB) of College of Health Sciences, Addis Ababa University. The health facilities provide breast cancer treatment like chemotherapy and surgery after pathologic confirmation. Among the seven facilities, Tikur Anbessa and St Paul’s hospitals are government hospitals; whereas, the rest (Betezata Hospital, Hallelujah Hospital, Legahar Clinic, MCH Hospital, and Landmark Hospital) are privately owned. Tikur Anbessa Specialized Hospital is the only health facility which provides radiotherapy service in Ethiopia [27].

### Study participants and sample size

The sample size was calculated using these assumptions: 95% confidence interval, margin of error (d) = 5%, prevalence of depression 40.3% [28] and 20% non-response rate. The final calculated sample size was 444. The eligibility criteria were all pathologically confirmed breast cancer patients who are above 18 years of age and who were on treatment in those selected health facilities in Addis Ababa, Ethiopia.

### Data collection and tools

Depression was assessed using a standard measurement scale called (PHQ-9). The PHQ-9, originally written in English, was translated to Amharic and was validated in Ethiopia and yielded high internal consistency [29]. MSPSS and pain assessment scores were used to assess patients’ social support and level of pain. The MSPSS scale was used with a view to identifying the participants’ perceived social support elements, developed by Gregory D Zimet (1988). The scale which evaluates the adequacy of social support received from three different sources in a subjective way consists of 12 items such as emotional support, source of comfort, sharing joy/ sorrow [30]. The three groups each of which has four items about the source of social support are family (3rd, 4th, 8th, and 11th items), friends (6th, 7th, 9th, and 12th items) and a special person (1st, 2nd, 5th, and 10th items). Each item is rated on a 7-point Likert scale. The 12-item scale uses a 7-point Likert type response format (1 = very strongly disagree, 7 = very strongly agree). Hence, higher scores indicate high social support [28].

Questions about sociodemographic (age, sex, educational background), behavioral (khat, alcohol, smoking history), clinical (stage, hormone receptor status, type of treatment) and psychosocial factors like history of violence, physical assault, family history of mental illness were developed after reviewing literatures and were adopted to our context. Eight BSc level trained nurses were recruited as data collectors and were trained on the objective of the study, data collection tools and interview techniques by the investigators. The principal investigator monitored the data quality and supervised the overall data collection process. After checking for completeness, the data was entered on Epi Data version 3.2.

### Data analysis procedures

After the data was entered and cleaned in Epi data software, it was transferred and analyzed using Statistical Package for Social Sciences (SPSS) version 25. Descriptive statistics like frequencies, mean and standard deviations were calculated based on the standard PHQ9 cut off points 0–4, 5–9, 10–14, 15–19 and ≥ 20 and considered as having minimal depression symptoms, minor depression, moderate depression (moderately severe) and major depression (severe) respectively. Then, the total depression score was dichotomized and those cancer patients who scored 10 and above were considered as having symptomatic depression. In order to identify associated factors, bivariate logistic regression analysis was done for each independent variable and significant variables were included in the final multiple logistic regression model using enter method. The crude and adjusted odds ratios (COR and AOR) with 95% CI were presented using tables and graphs. Since the assumption of normality was not fulfilled, the median and Interquartile Range (IQR) of overall MSPSS score and the three subcategories (Family, Friends and Significant Others) were calculated. Non-parametric tests like Mann-Whitney and Kruskal Wallis tests were computed to compare MSPSS score among depressed and non-depressed patients and across the different levels of depression.

## Results

### Sociodemographic characteristics of participants

Out of the total 428 women included in this study, 242 (56.5%) of them were married, 69 (16.1%) were widowed and 50 (11.7%) were divorced. The median age of the participants was 40 (IQR 15). Two thirds, 290 (67.8%), of the participants were Orthodox Christians, while 78 (18.2%) were Muslims. More than half 225 (52.6%) of them were living outside of Addis Ababa, and the majority 363 (84%) were treated at government hospitals. More than one fourth, 129 (30.1%), completed secondary education, and 83 (19.4%) were illiterate. Nearly half, 203 (47.4%), were housewives, and one fifth, 84 (19.6%), were government employees; close to one third, 126 (29.4%), of the women mentioned having financial crisis in their family in the last 1 year (Additional file 1: Table S1).

### Behavioral and clinical characteristics of participants

Regarding the impact of the disease on their life, 22 (6.9%) and 27 (6.3%) of the patients mentioned they encountered serious problem with their employer and family respectively. Forty-six (10.7%) of them also stated the disease affected their social activities. (Additional file 1: Table S2).

According to Tumor Node Metastasis (TNM) breast cancer classification, 184 (43.0%) and 163 (38.1%) had stage III and II breast cancers respectively. Even though hormone receptor status was checked only for few, 83 (19.4%), more than half 47 (56.6%) had Estrogen Receptor negative (ER –ve) breast cancer. Majority of the patients received chemotherapy 359 (83.9%) and surgery 377 (88.1%), while only few patients, 34 (7.9%), and 49 (11.4%) were given radiotherapy and hormonal therapy respectively. (Additional file 1: Table S3).

### Depression and social support

The prevalence of depression was 107/428 (25.0%) using the PHQ-9 score cutoff of ≥10. According to the PHQ-9 score categorization, 70 (65.4%), 30 (28.0%) and 7 (6.5%) had moderate, moderately severe and severe depression respectively. The participants’ MSPSS total scores was found to be high (70.35 ± 16.81), and the sub-dimensions of the MSPSS scale showed mean scores and standard deviations of 25.52 ± 4.97, 15.86 ± 9.44, and 24.0 ± 6.82 for family, friends, and significant others sub-dimensions respectively. There was a statistically significant association between social support and depression. Based on the Mann-Whitney test, depressed women were found to have lower mean rank score (MSPSS = 191.53) than non-depressed women (MSPSS = 221.51) (*P* = 0.027). Among the three sub dimensions of the MSPSS scale, statistically significant mean score difference was observed on social support received from significant others (SO). The result shows that depressed women had SO mean rank score of 190.11 while non-depressed women had 221.99, (*P* = 0.012). However, there is statistically significant mean rank score variation on social support received from family and friends. (Table 1)

**Table 1:** Multidimensional score for perceived social support among Breast cancer patients based on their depression status, Addis Ababa, Ethiopia, 2018

Variables	n (%)	Family	Friends	Significant other	Total MSPSS	*P*- value
Overall mean and SD	-	25.52±4.97	15.86±9.44	24.01±6.82	70.35±16.81	-
Overall median and IQR	-	28.00 IQR 3.00	16.00 IQR 20.00	28.00 IQR 5.00	76.00 IQR 18.00	-
Status of Depression						
Depressed (PHQ_9 ≥10	107 (25.0)	198.71	179.83	190.11*	191.53*	0.027
Not Depressed (PHQ_9 <10)	321 (75.0)	219.11	173.37	221.99	221.51	
Severity of Depression						
None (Minimal) (0-4)	171 (40.0)	235.67*	186.04	238.98*	241.30*	0.002
Minor (5-9)	150 (35.0)	200.35	158.72	202.73	199.08	
Moderate (10-14)	70 (16.4)	200.94	168.22	184.71	193.64	
Moderately severe (15-19)	30 (7.0)	204.67	206.33	207.02	203.07	
Severe (Major) (20-27)	7 (1.6)	150.93	189.60	171.64	121.00	

*Statistically significant difference on the mean score between the groups. *P*- value is only for overall MSPSS score

### Factors associated with depression

Age is one of the sociodemographic factors which was significantly associated with depression: as the age increased the risk of having depression decreased by 60–80%. Women who were above 30 years of age were less likely to have depression compared to women who were in the age category of 20–29 years [AOR = 0.40 (95% CI 0.17, 0.82)]. The other sociodemographic factor that was associated with depression was occupation: women who are merchants [AOR = 0.31 (95% CI 0.11, 0.86)] and who worked at private/non-governmental organizations [AOR = 0.36 (95% CI 0.14, 0.96)] were 70% less likely to have depression compared to women who are housewives. Those women who visited private health facilities were 0.32 times less likely to have depression as compared to the odds of women who were treated at government hospitals [AOR = 0.32 (95% CI 0.12, 0.82)].

Clinical factors like severity of pain and hormonal therapy were also found to have dose response association with depression. As the severity of pain increased the risk of depression also increased significantly: breast cancer patients who had moderate pain were nearly five times [AOR = 4.90 (95% CI 2.21, 10.82)] and those with severe pain were 13 times [AOR = 13.91 (95% CI 4.19, 46.31)] more likely to get depressed compared to breast cancer patients with no pain. Women who received hormonal therapy were 2.56 times more likely to have depression compared to women who didn’t receive hormonal therapy [AOR = 2.56 (95% CI 1.16, 5.64)]. Other factors like history of financial crisis, sexual assault and stage of cancer were not significantly associated with depression in this study. (Table 2)

**Table 2:** Multivariate logistic regression model for factors association with depression among Breast Cancer Patients in Addis Ababa, Ethiopia, 2018

Variables	Crude OR (95% CI)	Adjusted OR (95% CI)
Health facilities		
Government	1	1
Private	0.37 (0.17, 0.81)*	0.32 (0.12, 0.82)*
Age		
20-29	1	1
30-39	0.48 (0.23, 1.00)	0.40 (0.17, 0,82)*
40-49	0.49 (0.23, 1.05)	0.34 (0.14, 0.80)*
50-59	0.34 (0.14, 0.85)*	0.20 (0.07, 0.57)*
≥60	0.62 (0.27, 1.47)	0.26 (0.09, 0.73)*
Occupation		
Housewife	1	1
Merchant	0.38 (0.16, 0.90)*	0.31 (0.11, 0.86)*
Government	0.81 (0.46, 1.45)	0.93 (0.48, 1.82)
Private/NGO	0.54 (0.24, 1.17)	0.36 (0.14, 0.96)*
Farmer	1.22 (0.44, 3.40)	1.12 (0.34, 3.68)
other	0.76 (0.27, 2.17)	1.20 (0.37, 3.90)
Financial crisis		
No	1	1
Yes	1.63 (1.03, 2.60)*	1.05 (0.60, 1.83)
Sexual assault		
No	1	1
Yes	3.88 (1.02, 14.72)*	2.00 (0.18, 21.22)
Total problem^a^		
None	1	1
One	1.79 (0.90, 3.56)	1.83 (0.80, 4.22)
Two	3.71 (1.35, 10.21)*	4.72 (1.32, 16.7)*
Three and four	6.18 (2.18, 17.5)*	5.16 (1.49, 18.0)*
Severity of pain		
None (0)	1	1
Mild (1-3)	1.75 (0.74, 4.16)	1.18 (0.54, 2.57)
Moderate (4-6)	8.27 (3.50, 19.54)*	4.90 (2.21, 10.82)*
Severe (7-10)	17.51 (5.68, 54.13)*	13.91 (4.19, 46.31)*
Total score from SO		
	0.98 (0.94, 1.00)	0.97 (0.94, 1.00)
Final stage of cancer		
Stage I	1	1
Stage II	3.14 (0.90, 10.92)	0.91 (0.36, 2.34)
Stage III	3.70 (1.08, 12.73)*	0.84 (0.33, 2.13)
Stage IV	3.94 (0.99, 15.64)	0.84 (0.25, 2.86)
Hormonal therapy		
No	1	1
Yes	1.87 (1.00, 3.51)	2.56 (1.16, 5.64)*

*P value <0.05, significantly associated. *CI* confidence interval, *OR* odds ratio

^a^Problem/ impact of the disease on their family, work, social and spiritual life

## Discussion

In this study, the prevalence of depression among breast cancer patients was found to be 25%, which will have adverse impact on patients’ adherence to treatment, quality of life and overall survival. Similar prevalence of depression was reported from studies conducted in Malaysia (22%), Iraq (24.7%), Iran (26.7%), Qatar (27.7%), India (28%) and Morocco (26.9%) [10, 31–35]. However, our finding is lower than a study conducted in Tunisia which reported the prevalence of depression among cancer patients to be 48%, the explanation for such big difference could be the inclusion of women only aged 65 and above, who are demographically at higher risk of depression [36]. Similarly, high prevalence of depression was reported from Turkey (46%) [37], South Africa (36.6%) [38], Mexico (43%) [39] and Nigeria 40.3% [28] using Hospital Anxiety and Depression (HADs) tool, Center for Epidemiologic Studies Depression Scale (CESD) and Mini International Neuropsychiatric Interview (MINI) for assessment of depression respectively. In the two of the above listed studies, newly diagnosed breast cancer patients were included which can be the possible explanation for high prevalence of depression in those studies as significantly higher rates of depressive symptoms are expected in patients newly diagnosed with serious disease. This justification is also supported by a study from China which revealed Standardized Incidence Ratios (SIRs) of depression to be highest among newly diagnosed breast cancer patients [40].

In contrast, only 18% of breast cancer patients were found to be depressed according to a study done in Italy using Beck Depression Inventory (BDI) tool [41]. Similarly, a study conducted in Texas involving 430 breast cancer patients reported that only 16% of the patients scored PHQ8 > 10 and considered as depressed [42] compared to our study where 25% of women scored > 10. Even though these two studies have similar sample size, the observed variation might be due to the slight variation on the diagnostic tool used and the geographic difference between the two populations. In addition, the economic and sociocultural differences between the two populations might have contributed to this disparity.

According to the PHQ9 classification, 70 (16.4%), 30 (7.0%), 7 (1.6%) of total breast cancer patients were having moderate, moderately severe and severe depression respectively. This finding is slightly lower than a study done in Ethiopia which reported 22.5% moderate, 9.8% moderately severe and 4.6% for severe depression [43] among all cancer patients. However, mild depression was higher 150 (35%) in the current study compared to the above-mentioned study (28%). Likewise, a study done in Nigeria reported that 13 (39.4%), 12 (36.4%), 3 (9.1%) and 5 (15.1%) of breast cancer patients have minimal, mild, moderate and severe depression respectively [44]. This variation might probably occur as they studied all cancer patients while our study only included breast cancer patients. In addition, the smaller sample size in the Nigerian study might have inflated the prevalence of depression based on its severity level. Regarding prevalence of major depression, another study reported a prevalence of major depression among breast cancer patients to be 8.3% [45] whereas only 7 (1.6%) of the participants in our study had major depression. One explanation could be the use of different diagnostic tools used in the two studies. They used Mini International Neuropsychiatric Interview (MINI) tool for assessing depressive symptom while we used PHQ-9.

Regarding the different risk factors for depressive symptoms identified in this study, participants’ age was significantly associated with depression after controlling for other confounding factors. The risk of having depression decreased as the age increased, and women who were above 30 years of age were less likely to have depression compared to younger women who are found in the age category 20–29 years. This finding is also consistent with a study done in Italy, China and USA which reported psychological disorders including depression to be higher among younger patients [40, 41, 46]. Possible explanation for this is those women between 20 and 29 years of age were less likely to be married or had desired number of children. They are also more likely to refuse mastectomy and may suffer from fear of death at young age, which will predispose them to greater psychological distress and depression.

On the contrary, a recent study done among African American population in USA found that older age increased the risk of having depression compared to the younger age in women with breast cancer [47]. Another study done in Lithuania also reported that breast cancer patients over age 55 years had a 2.25 times greater risk of suffering from depression compared to those who were younger [48].

Pain was found to be significantly associated with depression, and as severity of pain increased the risk of having depression also increased among the current study participants. This finding is consistent with reports from studies conducted in Taiwan, Tunisia and Ethiopia [36, 43, 45]. This suggests that pain management is very crucial for breast cancer patients in the prevention of depression and other psychological co-morbidities.

Several studies reported that stage of the cancer is strongly associated with depression and breast cancer patients with advanced stage had higher risk of depression as compared to those with early stage cancer [43, 45, 49]. But in this study, the significant association found between depression and stage of cancer during the bivariate analysis was not significant after adjusting for other confounding factors.

In this study, hormonal therapy increased risk of having depression. Depression is one of the side effects of hormonal drugs including Tamoxifen, which could account for the increased prevalence of depression in patients treated with hormonal therapy [50]. This finding suggests that attention should be given for breast cancer patients especially for those who are on hormonal therapy. Despite what might be expected, there is a study which uncovered as hormonal treatment is protective against depression [45].

In this study, the overall perceived social support was found to be high (70 ± 16.81) compared to a study done in Turkey (57.41 ± 13.97) [51]. This variation might be due to the strong sociocultural practices and social bonding in Ethiopia. From the three domains of the social support scale, the highest sub dimensional social support scale belongs to family, followed by significant others and the lowest was from friends. This finding is consistent with previously conducted studies in Turkey and Qatar [33, 51].

The result of this study showed that there is significant association between social support and depression. Other studies also indicated that social support was lower among breast cancer patients with depression [49, 52], and family support was reported to be negatively associated with depression among breast cancer patients in China and Qatar [45, 53].

The main limitation of this study lies on its cross-sectional nature which makes it difficult to conclude on the direction of association between social support and depression. Moreover, only those breast cancer patients who visited health facilities were included in the study and the burden of depression cannot be generalized for all breast cancer patients since we didn’t include those who stayed at home. Strengths of the present study include large sample size, inclusion of almost all health facilities in Addis Ababa which actively provide cancer care and its focus on one of the understudied areas in cancer care in Ethiopia. In addition, a standard and validated tool was used to measure depression among the study participants.

## Conclusion

This study found that one in four breast cancer patients had depression, and the overall social support received from family, friends and significant others was high. However, the overall social support score was also found to be significantly different among depressed and non-depressed patients. The social support score declined as the severity of depression increased. Age, occupation, type of health facility treated, severity of pain, hormonal therapy and having problem with employer or family were identified factors that were associated with depression. Hence, we recommend integration of screening for depression and psychosocial service provision in the routine cancer care in Ethiopia.

## Additional file

Additional file 1: **Table S1.** Sociodemographic Characteristics of Breast Cancer Patients, Addis Ababa, Ethiopia, 2018. **Table S2.** Behavioral Characteristics of Breast Cancer Patients in Addis Ababa, Ethiopia, 2018. **Table S3.** Clinical Characteristics of Breast cancer patients in Addis Ababa, Ethiopia, 2018. (DOCX 32 kb)

## Data Availability

The datasets used and/or analyzed during the current study are available from the corresponding author on reasonable request.

## Abbreviations

AOR: Adjusted Odds Ratio
BDI: Beck Depression Inventory
CES-D: Center for Epidemiological Studies for Depression
COR: Crude Odds Ratio
DSM-IV: Diagnostic and Statistical Manual of Mental Disorders
ER: Estrogen Receptor
HADS: Hospital Anxiety and Depression Scale
IRB: Institutional Review Board
MFS: Metastasized Free Survival
MINI: Mini International Neuropsychiatric
MSPSS: Multidimensional Scale of Perceived Social Support
NGO: Non-Governmental Organizations
PHQ9: Patient Health Questionnaire 9
REC: Research Ethics Committee
SID: Standardized Incidence Ratios
SPSS: Statistical Package for Social Sciences
TNM: Tumor Nodes Metastasis
USA: United states of America
WHO: World Health Organization
