# Supplementary material for: Depression and social support among breast cancer patients in Addis Ababa, Ethiopia
Source: BMC Cancer. 2019 Aug 27;19:836. doi: 10.1186/s12885-019-6007-4 (PMC6712811; doi:10.1186/s12885-019-6007-4)
Supplement: Supplementary file 1 — Table S1. Sociodemographic Characteristics of Breast Cancer Patients, Addis Ababa, Ethiopia, 2018. Table S2. Behavioral Characteristics of Breast Cancer Patients in Addis Ababa, Ethiopia, 2018. Table S3. Clinical Characteristics of Breast cancer patients in Addis Ababa, Ethiopia, 2018. (DOCX 32 kb) [file 12885_2019_6007_MOESM1_ESM.docx]

Table S1: Sociodemographic Characteristics of Breast Cancer Patients, Addis Ababa, Ethiopia, 2018.

| **Variables** |  |  | | | **n = 428** | **Percent (%)** |
| --- | --- | --- | --- | --- | --- | --- |
| **Categorized age** | 20-29  30-39  40-49  50-59  ≥60 |  | | | 41  157  113  61  56 | 9.6  36.7  26.4  14.3  13.1 |
| **Type of health facility** | Government  Private |  | | | 363  65 | 84.8  15.2 |
|  |  |  | | |  |  |
| **Educational level** | Illiterate  Read & write  Primary  Secondary  Tertiary |  | | | 88  26  83  129  102 | 20.6  6.1  19.4  30.1  23.8 |
| **Religion** | Orthodox  Catholic  Protestant  Muslim |  | | | 290  5  55  78 | 67.8  1.2  12.9  18.2 |
| **Residence** | Inside Addis  Outside of Addis | |  | | 203  225 | 47.4  52.6 |
| **Marital Status** | Married  Cohabitated  Divorced  Widowed  Single |  | | | 242  19  50  69  48 | 56.5  4.4  11.7  16.1  11.2 |
| **Husband’s occupation (n=261)** | Nothing  Merchant  Government employee  Private/ NGO  Farmer  Others* | | |  | 23  48  75  64  30  21 | 8.8  18.4  28.7  24.5  11.5  8.0 |
| **Husband’s educational level (n=261)** | Illiterate  Read and write  Primary  Secondary  Tertiary | | |  | 35  13  38  78  97 | 13.4  5.0  14.6  29.9  37.1 |
| **Occupation** | Housewife  Merchant  Government employee  Private /NGO^a^  Farmer  other | | |  | 203  52  84  50  18  21 | 47.4  12.1  19.6  11.7  4.2  4.9 |
| **Financial crisis** | No  Yes | | |  | 102  126 | 70.6  29.4 |

*Others include: laundry, retired, husbandry, pension. ^a^NGO – nongovernmental organizations

**Table S2: Behavioral Characteristics of Breast Cancer Patients in Addis Ababa, Ethiopia, 2018.**

| **Variables** |  |  | | **n** | | | **Percent (%)** |
| --- | --- | --- | --- | --- | --- | --- | --- |
| **Physical assault** | No  Yes |  | | 413  15 | | | 96.5  3.5 |
| **Partner violence (n=285)** | No  Yes |  | | 269  16 | | | 94.4  5.6 |
| **Sexual assault** | No  Yes |  | | 419  9 | | | 97.9  2.1 |
| **Death of family member** | No  Yes |  | | 284  144 | | | 66.4  33.6 |
| **Khat** | No  Yes |  | | 386  42 | | | 90.2  9.8 |
| **Smoking** | No  Yes |  | | 424  4 | | | 99.1  0.9 |
| **Alcohol** | No  Yes |  | | 350  78 | | | 81.8  18.2 |
| **Chronic disease** | No  Yes |  | | 345  83 | | | 80.6  19.4 |
| **Know about their disease** | Breast cancer  Breast tumor and inflammation  Don’t know | |  | 354  61  12 | | | 82.9  14.3  2.8 |
| **History of mental illness** | No  Yes | | | | 419  9 |  | 97.9  2.1 |
| **Family history of mental illness** | No  Yes | |  | 398  30 | | | 93.0  7.0 |
| **Problem on social activities** | No  Yes | |  | 382  46 | | | 89.3  10.7 |
| **Problem with employer(n=317)** | No  Yes | |  | 295  22 | | | 93.1  6.9 |
| **Problem on spiritual life** | No  Yes | |  | 396  32 | | | 92.5  7.5 |
| Problem with family | No  Yes | |  | 401  27 | | | 93.7  6.3 |

*Table 1 will be cited after sociodemographic characteristics subsection of the result.*

*Table 2 &3 will be cited immediately after description of behavioral and clinical characteristics of participants*

Table S3: Clinical Characteristics of Breast cancer patients in Addis Ababa, Ethiopia, 2018.

| **Variables** | | |  |  | | **n** | **Percent (%)** |
| --- | --- | --- | --- | --- | --- | --- | --- |
| **Severity of pain** | | | None  Mild  Moderate  Severe |  | | 76  212  114  25 | 17.8  49.5  26.6  5.8 |
| **Stage of cancer(n=417)** | | Stage I  Stage II  Stage III  Stage IV | |  | | 32  163  184  38 | 7.5  38.1  43.0  8.9 |
| **Duration of treatment since diagnosis (n=381)** | <=1 year  2-3 years  4-5 years  >5 years | | | |  | 240  67  12  62 | 56.1  15.7  2.8  14.5 |
| **Type of breast cancer** | | ER-ve  ER+ve  NA | |  | | 47  36  345 | 11.0  8.4  80.6 |
| **Chemotherapy** | | No  Yes | |  | | 63  359 | 14.7  83.9 |
| **Surgery** | | No  Yes | |  | | 45  377 | 10.5  88.1 |
| **Radiotherapy** | | No  Yes | |  | | 388  34 | 90.7  7.9 |
| **Hormonal therapy** | | No  Yes | |  | | 372  49 | 86.9  11.4 |
